# The Involvement of the McsB Arginine Kinase in Clp-Dependent Degradation of the MgsR Regulator in *Bacillus subtilis*

**DOI:** 10.3389/fmicb.2020.00900

**Published:** 2020-05-12

**Authors:** Lars Lilge, Alexander Reder, Frank Tippmann, Friedrich Morgenroth, Janice Grohmann, Dörte Becher, Katharina Riedel, Uwe Völker, Michael Hecker, Ulf Gerth

**Affiliations:** ^1^Institute of Microbiology, University of Greifswald, Greifswald, Germany; ^2^Interfaculty Institute for Genetics and Functional Genomics, University of Greifswald, Greifswald, Germany; ^3^Institute of Marine Biotechnology, Greifswald, Germany

**Keywords:** Clp proteolysis, McsB arginine kinase, MgsR degradation, arginine phosphorylation, MgsR activity

## Abstract

Regulated ATP-dependent proteolysis is a common feature of developmental processes and plays also a crucial role during environmental perturbations such as stress and starvation. The *Bacillus subtilis* MgsR regulator controls a subregulon within the stress- and stationary phase σ^B^ regulon. After ethanol exposition and a short time-window of activity, MgsR is ClpXP-dependently degraded with a half-life of approximately 6 min. Surprisingly, a protein interaction analysis with MgsR revealed an association with the McsB arginine kinase and an *in vivo* degradation assay confirmed a strong impact of McsB on MgsR degradation. *In vitro* phosphorylation experiments with arginine (R) by lysine (K) substitutions in McsB and its activator McsA unraveled all R residues, which are essentially needed for the arginine kinase reaction. Subsequently, site directed mutagenesis of the MgsR substrate was used to substitute all arginine residues with glutamate (R-E) to mimic arginine phosphorylation and to test their influence on MgsR degradation *in vivo*. It turned out, that especially the R33E and R94/95E residues (RRPI motif), the latter are adjacently located to the two redox-sensitive cysteines in a 3D model, have the potential to accelerate MgsR degradation. These results imply that selective arginine phosphorylation may have favorable effects for Clp dependent degradation of short-living regulatory proteins. We speculate that in addition to its kinase activity and adaptor function for the ClpC ATPase, McsB might also serve as a proteolytic adaptor for the ClpX ATPase in the degradation mechanism of MgsR.

## Introduction

One of the most remarkable defense strategies of *Bacillus subtilis* against stress and starvation is the general stress response controlled by the alternative sigma factor σ^B^ resulting in a non-specific and multiple stress resistance (Hecker et al., 2007). σ^B^ as master regulator recognizes a particular promoter structure (Boylan et al., 1991) and controls more than 200 genes, which mediate a cross-protection against different stress conditions (Hecker et al., 2007). The activation of σ^B^ is based on different physical stress factors such as low temperature (Brigulla et al., 2003) or heat (Benson and Haldenwang, 1993; Boylan et al., 1993), salt (Boylan et al., 1993; Voelker et al., 1995), ethanol (Boylan et al., 1993), and acid shock as well as oxygen limitation (Voelker et al., 1995). In addition σ^B^ is activated in the presence of nitric oxide (NO) or sodium nitroprusside (SNP) (Moore et al., 2004), cell wall stress inducing reagents like vancomycin or bacitracin (Mascher et al., 2003) and by components decreasing the intracellular ATP pool like carbonyl cyanide m-chlorophenylhydrazone (CCCP) (Voelker et al., 1995; Alper et al., 1996), azide and mycophenolic acid (Zhang and Haldenwang, 2005). Furthermore, the general stress response is also mediated by nutrient starvations like glucose and phosphate (Voelker et al., 1995) as well as by blue light radiation (Gaidenko et al., 2006).

Sophisticatedly, the σ^B^ regulon includes the redox-sensitive modulators MgsR and Spx for a fine-tuned expression of genes involved in secondarily induced oxidative or thiol-specific stress (Nakano et al., 2005; Reder et al., 2008, 2012). These paralogous transcriptional regulators are members of ArsC family and both contain a conserved redox-sensitive CxxC motif. Disulfide bond formation after oxidative stress (Nakano et al., 2005; Reder et al., 2012) results in differential gene regulation of approximately 70 genes for MgsR (Reder et al., 2008) and 300 genes for Spx (Nakano et al., 2003a). In contrast to *mgsR*, which is exclusively σ^B^ dependently induced and positively autoregulated (Reder et al., 2008), the *yjbC-spx* operon is subjected to a complex regulatory network. Transcription is initiated by a variety of sigma factors (σ^B^, σ^M^, σ^w^, and σ^A^) (Antelmann et al., 2000; Cao et al., 2002; Antelmann and Helmann, 2011), an intergenic σ^A^ promoter (*spx* P3) (Leelakriangsak and Zuber, 2007) but also prevented by the repressors PerR and YodB (Leelakriangsak et al., 2007). Ensuring an organized and timely limited action, MgsR and Spx are subjected to proteolysis that is commonly performed by ClpXP (Nakano et al., 2003b; Reder et al., 2012) and subordinated/partially by ClpC (Nakano et al., 2002, 2003b; Reder et al., 2012). It was suggested, that during oxidative stress the sensitive zinc-binding domain (ZBD) of ClpX falls apart leading to deactivation and aggregation of ClpX and its adaptor protein YjbH, a condition promoting the stabilization and accumulation of oxidized and activated Spx (Zhang and Zuber, 2007). Nonetheless, differences exist between MgsR and Spx degradation. The adaptor protein YjbH appeared to influence only Spx degradation, whereas MgsR turnover was unaffected by a deletion of the *yjbH* gene (Larsson et al., 2007; Reder et al., 2012).

Besides the use of adaptor proteins, also the attachment of functional groups such as phosphate groups to specific amino acid residues seemed to accelerate the degradation of target proteins (Trentini et al., 2016). The versatility and reversibility of this post-translational modification (PTM) are key aspects for the widespread existence of protein kinases and phosphatases (Hunter, 2012; Cousin et al., 2013). Thereby complexity increases by a sizeable number of phosphorylatable amino acid side chains (Macek and Mijakovic, 2011). Interestingly and in addition to a considerable number of O-phosphorylating serine/threonine and tyrosine kinases, *B. subtilis* also encodes also the *N*-phosphorylating arginine kinase McsB (Fuhrmann et al., 2009), which appears to influence the activity and stability of selected proteins (Kirstein et al., 2005, 2007; Fuhrmann et al., 2009; Elsholz et al., 2011a, 2012; Trentini et al., 2016).

A complex regulatory network controls McsB expression and activity under different physiological conditions (Elsholz et al., 2017). The transcriptional repressor CtsR controls *mcsB* gene expression during standard growth conditions (Krüger and Hecker, 1998). Additionally, protein–protein interaction of McsB with the ClpC ATPase leads to inactivation of the McsB kinase (Kirstein et al., 2005, 2007; Elsholz et al., 2011a). On the one hand, kinase activity is stimulated by the interaction partner McsA (Kirstein et al., 2005, 2007; Elsholz et al., 2010). In this state, McsB has the ability to inactivate CtsR by phosphorylation, promoting its degradation by ClpCP and ClpEP (Kirstein et al., 2005, 2007; Miethke et al., 2006; Elsholz et al., 2010, 2011a). On the other hand, thiol-specific stress oxidizes the second McsA Zn-finger and abrogates the interaction between McsA and McsB, liberated McsB in turn inactivates CtsR (Elsholz et al., 2011b).

As expected, *B. subtilis* also encodes an antagonistic player, the arginine phosphatase YwlE, which restores the arginine-dephospho-state of proteins and inhibits McsB activation by autophosphorylation (Kirstein et al., 2005, 2007; Elsholz et al., 2011a, 2012; Fuhrmann et al., 2013, 2016). However, YwlE is also a redox-sensitive enzyme that is inactivated by oxidation of cysteine residues in its active center resulting in partial activation of McsB kinase (Chiarugi and Cirri, 2003; Fuhrmann et al., 2016).

In this active state, McsB acts as an adaptor protein of ClpC, stimulating the ATPase activity by site-specific phosphorylation (Kirstein et al., 2007; Elsholz et al., 2012) and also seems to label proteins for ClpCP-mediated degradation (Trentini et al., 2016). However, *in vivo* experiments showed that heat-inactivated CtsR appeared to be degraded by ClpCP independently of the repressor’s phospho-state (Elsholz et al., 2010).

So far, more than 150 proteins were identified as putative targets of McsB (Fuhrmann et al., 2009; Elsholz et al., 2012; Schmidt et al., 2013; Trentini et al., 2014, 2016), however, the exact physiological role of the arginine phosphorylation (arg-P) of the target proteins still remains obscure. In general, arginine represents a key amino acid for protein–protein interaction (Bogan and Thorn, 1998) and is one of the most important residues for protein-DNA contact (Schneider et al., 2013).

In this report, we present new insights into targeted proteolysis of *B. subtilis* MgsR. We identified a protein interaction between MgsR and arginine kinase McsB during ethanol stress *in vivo*. The impact of McsB kinase on the turnover of MgsR during stress conditions was analyzed by radioactive pulse-chase labeling and immunoprecipitation. Essential McsB arginine residues were identified by exchange mutagenesis and radioactive *in vitro* phosphorylation. In addition, site-directed mutagenesis was performed on selected MgsR arginine residues and both, stability and regulatory activity of these mutant forms was monitored simultaneously *in vivo*. Based on these findings, we were able to characterize the impact of specific arg-P mimics on MgsR stability and activity during general stress response.

## Results

### Putative Interaction Partner of MgsR

**FIGURE 1:**
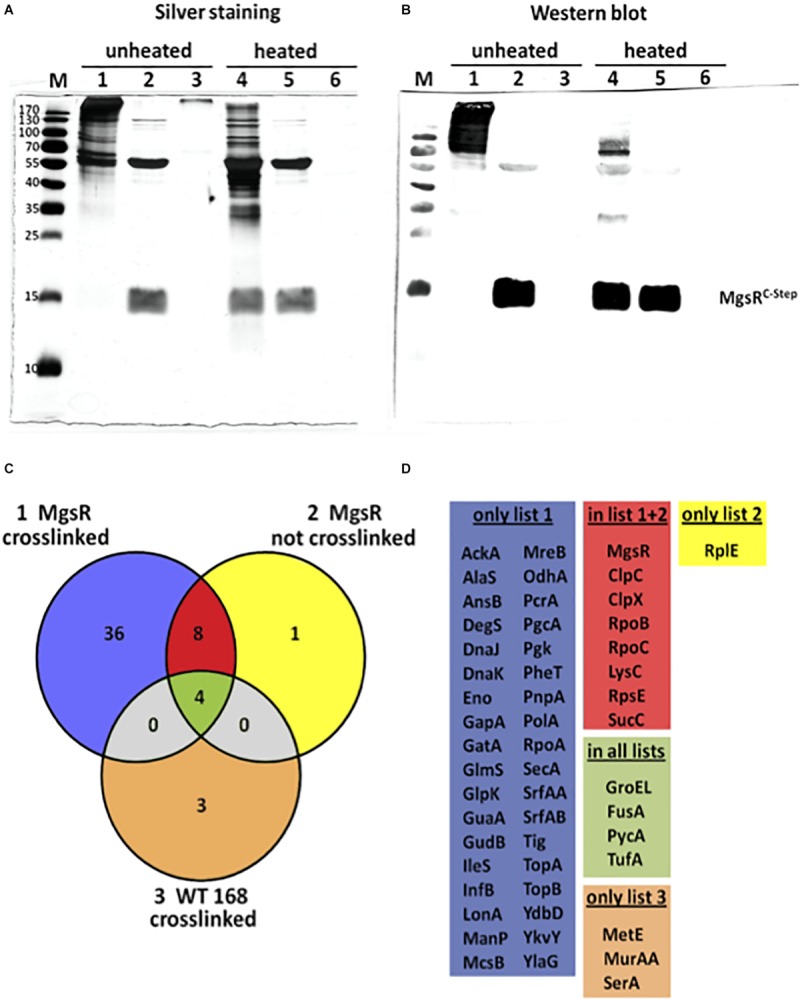
Evaluation of the SPINE assay by SDS-PAGE and mass spectrometry. Analysis of three different protein samples by **(A)** silver staining and **(B)** Western blotting with MgsR specific antibodies; samples 1 and 4 represent MgsR^C–Strep^ protein that was purified upon formaldehyde cross-linking; samples 2 and 5 shows MgsR^C–Strep^ that was purified without cross-linking and samples 3 and 6 represent proteins purified from the *B. subtilis* wild-type after formaldehyde cross-linking that serve as a negative control. Samples were mixed with Laemmli-sample buffer and either loaded directly on the SDS-PAGE (non-heated – samples 1 to 3) or incubated at 95°C for 20 min (heated – samples 4 to 6) to reverse the cross-linking prior loading. M = molecular weight marker as indicated in kDa. **(C)** Color coded Venn diagram that schematically summarizes the number of all identified proteins from the three approaches and their overlapping sets. **(D)** Listings of the identified proteins that were grouped into the five sets as indicated by the Venn diagram; list 1 (blue) comprises proteins solely identified from crosslinked MgsR^C–Strep^ purifications; list 2 (yellow) includes proteins exclusively identified from non-crosslinked MgsR^C–Strep^ samples; list 3 (ocher) is a set of proteins only identified from crosslinked wild-type cultures; overlap of lists 1 and 2 (red) are proteins identified from crosslinked and non-crosslinked MgsR^C–Strep^ purifications and common to all lists (pale green) are proteins identified from all samples representing false positives.

To identify potential *in vivo* interaction partners of MgsR, a Strep–protein interaction experiment was carried out called SPINE (Herzberg et al., 2007). Therefore, crosslinking by formaldehyde was combined with Strep-tag protein purification. For this purpose, a chromosomal copy of *mgsR*^C–Strep^ with its original regulatory upstream sequence (200 bp) was integrated into *amyE* locus in Δ*mgsR* mutant background. Thus, the *mgsR*^C–Strep^ copy was under transcriptional control of its own promoter and remained fully functional observed for MgsR^C–Strep^ itself as well as the target gene products YdbD and YhxD (Reder et al., 2008) after exposure to 4% ethanol stress (data not shown). In this way, MgsR expression is under normal physiological conditions and potential pleiotropic effects caused by otherwise ectopic overproduction of the protein were avoided. In accordance with previous results that demonstrated a maximal cellular concentration of MgsR as well as its activity about 10 min after exposure of 4% ethanol stress (Reder et al., 2008), also formaldehyde was added to the cultures 10 min after induction of the mgsR^C–Strep^ construct by 4% ethanol. Aliquots obtained from crosslinked and non-crosslinked Strep-tag purifications as well as a cross linked negative control (wild-type) were analyzed by SDS-PAGE combined with silver staining (Figure 1A) as well as Western blot analysis using MgsR specific antibodies (Figure 1B). Lane 1 of the silver stained gel as well as lane 1 of the Western blot demonstrate that the purified proteins in the unheated samples are still present in macromolecular complexes and that MgsR is completely bound within these complexes. Furthermore, lane 4 of both approaches point out that crosslinking of these complexes can be almost fully reversed by heating the samples. In contrast, the four aliquots of purified non-crosslinked MgsR^C–Strep^ do not differ from each other with respect to unheated and heated samples in the silver stained gel (Figure 1A, lanes 2 and 5) as well as the Western blot (Figure 1B, lanes 2 and 5) demonstrating that MgsR is not bound in any covalent complexes. Nevertheless, it is striking that one major protein band was co-purified with MgsR^C–Strep^ that could be identified later as the chaperonin GroEL. Crosslinked and “purified” proteins of the *B. subtilis* wild-type 168 were included as negative control (Figures 1A,B, lanes 3 and 6). Next, we carried out a mass spectrometric (MS) analysis to identify the proteins from all three purification approaches, aiming to decrease the number of potential interaction partners and to distinguish relevant from non-specific interactions. Results of the MS-approach are summarized in a Venn diagram (Figure 1C) and are allocated in five different groups of identified proteins according to the overlapping sets (Figure 1D). Interestingly, all but one of the 13 proteins identified from non-crosslinked MgsR^C–Strep^ samples are either part of the crosslinked MgsR^C–Strep^ or the negative control group (yellow) (Figures 1C,D). At least eight of these 13 proteins are present only in crosslinked and non-crosslinked samples (red) tempting to speculate whether an interaction of these proteins with MgsR is specific and strong enough to withstand the purification procedure without formaldehyde crosslinking, sufficient for identification by our highly sensitive MS-approach. This group includes the RNA polymerase subunits RpoB and RpoC. With respect to the fact that Spx was shown to primary target the *C*-terminal domains of RpoA (Nakano et al., 2003a; Newberry et al., 2005) the α-subunit was also identified from the crosslinked MgsR samples (Figure 1D). Furthermore, the ATPase subunits ClpC and ClpX were identified from both approaches pointing out their involvement in degradation of MgsR. Although previous studies disproved an impact of LonA on MgsR stability (Reder et al., 2012), a weak interaction seems to be detectable indicating a slight proteolytic influence in the background of ClpXP and ClpCP. Most strikingly, also arginine kinase McsB was detected as crosslinked interaction partner. Considering previous results that arg-P decreases protein stability and functionality (Kirstein et al., 2005; Trentini et al., 2016), MgsR turnover and activity could be influenced by McsB. Taken together, SPINE approach narrowed the amount of potential MgsR interaction partners to a promising and manageable number of 44 candidate proteins.

### Half-Life Determination of MgsR *in vivo* in Different Genetic Backgrounds

**FIGURE 2:**
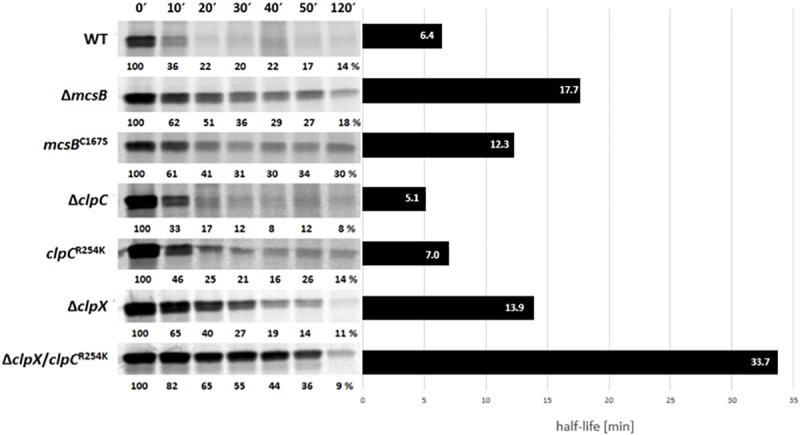
Immunoprecipitation of radiolabeled MgsR from wild type and different isogenic mutant strains were performed after induction with 4% (v/v) ethanol and radioactive pulse-chase-labeling with ^35^*S*-*L*-methionine. Immediately after addition of non-radioactive methionine, the first sample was taken (0′) and 10, 20, 30, 40, 50, and 120 min later. The calculated half-life of MgsR are visualized in the column diagram on the right side.

The impact of the arginine kinase McsB on stability of MgsR was determined after an induction of secondary oxidative stress by addition of 4% ethanol. Therefore, a radioactive pulse-chase labeling approach combined with immunoprecipitation was performed and a MgsR half-life in *B. subtilis* wild type was confirmed with 6.4 min as described previously by Reder et al. (2012). The autoradiogram depicts a remaining basal protein level of approximately 10–20% after 20 min indicating that a sub fraction persists with a higher stability (Figure 2) (Reder et al., 2012).

In the next step, MgsR stability was analyzed in both, a Δ*mcsB* deletion as well as a kinase inactive *mcsB*^C167S^ mutant (Kirstein et al., 2005) that resulted in a significant increase of MgsR half-life (Figure 2). The higher basal level of MgsR already indicates that arg-P could be an important trigger for protein degradation. Interestingly, the sole inactivation of the McsB kinase function in the *mcsB^C167S^* mutant also resulted in an extended MgsR half-life (12.3 min) compared to the wild type, but was also slightly shortened in comparison to the Δ*mcsB* deletion mutant (17.7 min). These results indicate that (i) the McsB kinase activity plays an important role for MgsR destabilization but also that (ii) the McsB protein itself is needed for proper MgsR degradation. The difference in half-life extension observed in both mutant strains prompted us to speculate that kinase activity and adaptor protein function of McsB are two distinguishable modes of operation.

Interestingly, a deletion of McsB causes a comparable impact such as a deletion of *clpX* does the main factor for MgsR degradation (Figure 2) (Reder et al., 2012). In contrast, a deletion of ClpC and a non-activatable ClpC^R254K^ background (Figure 2) (Elsholz et al., 2012) show no effect on MgsR half-life. These results further allow the speculation that McsB may take action as an adaptor protein of ClpX for MgsR degradation.

### Identification of Arginine Residues Required for McsB-Kinase Activity *in vitro*

**FIGURE 3:**
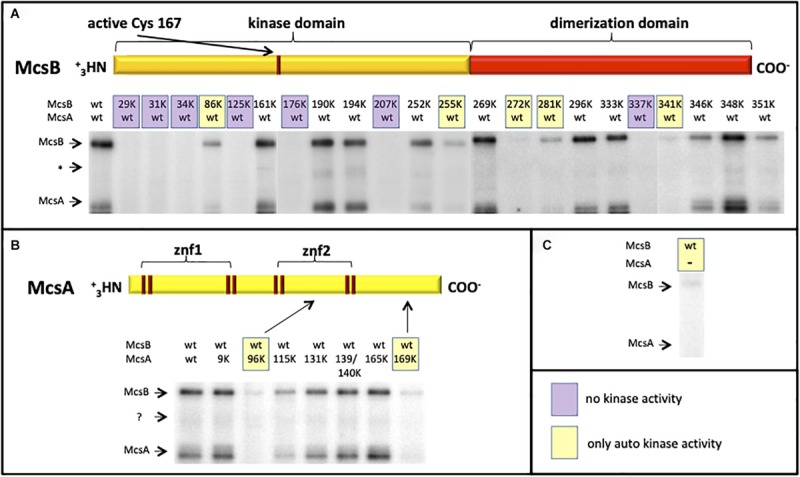
Radioactive (γ−−^32^P) ATP *in vitro* phosphorylation assay with different R-K point-mutated and purified McsB and McsA proteins. The autoradiograms depict the phosphorylation status of wild type and different McsB **(A)** and McsA **(B)** point mutated proteins. For a direct comparison the weak McsB auto-kinase activity (in absence of McsA) is shown on the right **(C)**. Stimulated and full kinase activity can be observed with the wild-type proteins and selected mutant proteins **(A, B)**. Some variants show no kinase (shaded violet; **A**) or only auto-kinase activity (shaded yellow; **A–C**). ^∗^Low abundant contaminant.

The 3-D structure was recently solved and it was shown that the McsB phosphagen-like phosphotransferase domain is structurally adapted to target protein substrates and accompanied by a novel arg-P-binding domain that allosterically controls protein kinase activity (Suskiewicz et al., 2019). In addition to a weak auto-phosphorylation activity, McsB can be fully activated by McsA (Kirstein et al., 2005), to phosphorylate other targets such as ClpC, CtsR, MgsR and approximately 200 different proteins (Elsholz et al., 2012; Schmidt et al., 2013; Trentini et al., 2014, 2016). McsB is a structurally unique arginine kinase, where the first and bigger part contains a guanidino-phosphotransferase domain and the second smaller part a dimerization domain with a pArg-binding pocket (Suskiewicz et al., 2019). A multiple McsB sequence alignment with selected members of low GC Gram+ bacteria shows highly conserved arginine residues (Supplementary Figure S1). All conserved arginine residues in McsB as well as in McsA were substituted with lysine residues and the mutant proteins were tested in a radioactive γ-^32^P-ATP *in vitro* phosphorylation assay to identify arginines, which are crucial for the McsB kinase reaction. It turned out that especially arginine residues from the very *N*-terminus (R29K, R31K, and R34K), three arginine residues from the middle kinase domain (R125K, R176K, and R207K) and one from the *C*-terminus (R337K) are essential for the McsB kinase (Figure 3), since no radioactive phosphoprotein signal was obtained for these McsB point mutants. All the above mentioned arginine residues belong to the McsB active site with the exception of R31 and R337 (Suskiewicz et al., 2019). Notice that the mutated arginine residues at position R337K and R341K from *C*-terminal pArg-binding pocket R^337^DXXR^341^A motif (Suskiewicz et al., 2019) deliver no or almost no McsB phospho-signal underpinning the essentiality of this proposed pocket for the proper functioning and allosteric control of the McsB enzymatic activity. Potentially, this site is also involved in McsA binding via the second zinc finger (Elsholz et al., 2011b). A weak radioactive phosphoprotein-signal was detected for the McsB (R86K, R255K, R272K, R281K, and R341K) and McsA point mutants (R96K and R169K) showing that autophosphorylation of McsB is still possible, but the stimulatory effect of McsA vanished. Interestingly, McsA R96 is located within the loop of the second zinc finger and R169 at the very *C*-terminus of McsA, both arginine residues seem important for the interaction and stimulation of the McsB kinase. All other arginine-lysine substitutions in McsA (R9K, R115K, R131K, R139/140K, and R165K) do not influence the stimulatory effect of McsA toward the McsB arginine kinase.

### Phospho-Mimicking of Putative MgsR Phospho-Sites

To check whether specific MgsR arginine residues are involved in targeted proteolysis, a strain was constructed, in which MgsR autoregulation was completely abolished (Reder et al., 2012) and expression exclusively depend on the presence of externally added xylose (Supplementary Figure S3). In this way, amino acid substitutions in MgsR do not interfere with its own expression. For this purpose, the pX vector (Kim et al., 1996) was used to fuse *mgsR* and its original upstream sequence (+34 bp) including the transcriptional start site (Reder et al., 2008) with the *xylA* promoter, which is under negative control by the XylR repressor.

**FIGURE 4:**
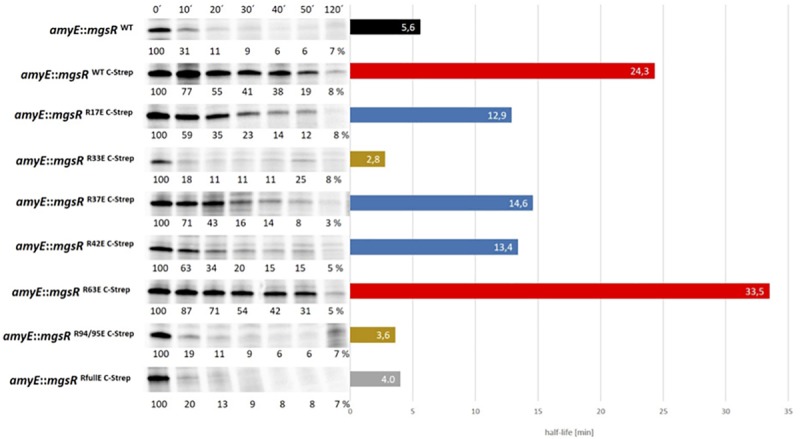
Degradation kinetics of different R/E substituted MgsR^C–Strep^ versions were determined by ^35^*S*-methionine pulse-chase radioimmunoprecipitation. Cells were treated with 0.3% (w/v) xylose, 4% (v/v) ethanol, and labeled with 25 μCi/ml ^35^*S*-methionine at an OD_500 nm_ of 0.4. A total of 10 min after the radioactive pulse, reaction was chased by ^32^*S*-*L*-methionine and the first sample (0′) was taken. Further samples were collected after 10, 20, 30, 40, 50, and 120 min. The calculated half-life is visualized adjacent to the MgsR degradation kinetics, whereas the black bar depicts the unmodified wild type MgsR. Red bars show MgsR-strep and the R63E mutant with significantly extended half-life. Blue bars depict R17E, R37E, and R42E mutants with a two-fold decreased half-life and ochre bars represent the R33E and R94/95E mutants with similar half-life such as the R/E full mutant (gray bar).

**FIGURE 5:**
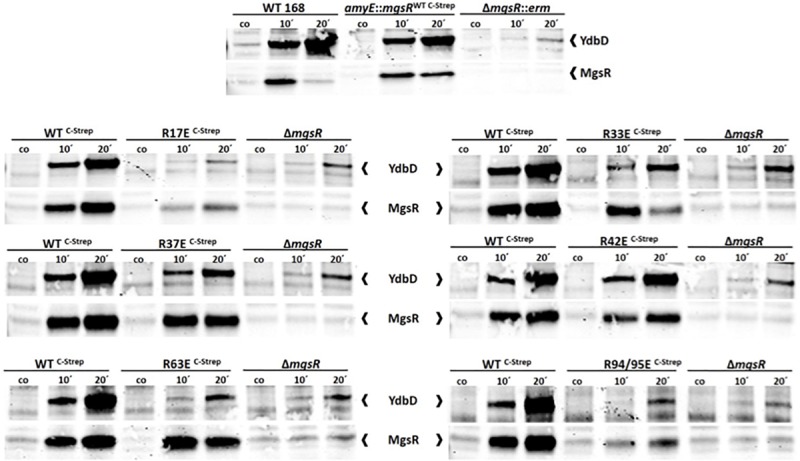
Verification of MgsR activity as transcriptional activator by Western Blot analyses. **(A)** Sampling for the detection of both, YdbD and MgsR, was performed 10 and 20 min after stress exposure to 4% (v/v) ethanol and 0.3% (v/v) xylose mediated *mgsR* induction, whereas a control sample (co or 0′) was taken immediately before stress. MgsR and MgsR-dependent YdbD induction pattern of *Bacillus subtilis* wild type 168 cells (positive control) was compared with the kinetic of BLL35 (*amyE*:*mgsR*^WT C–Strep^) and the Δ*mgsR* deletion mutant (negative control). **(B)** Induction kinetics of BLL34 was compared with the respective R/E point mutants of the Strep-tagged MgsR version and Δ*mgsR*.

Based on this system, substitutions with negatively charged glutamate residues were introduced to mimic arg-P of MgsR. According to Trentini et al. (2016), arg-P marks proteins for ClpCP dependent proteolysis, therefore R/E-substitutions should destabilize MgsR resulting in a faster degradation. However, fast vanishing R/E-MgsR-species cause detection difficulties. By chance we realized that the addition of a Strep-tag to the *C*-terminus of MgsR resulted in a prolonged half-life, a similar effect was observed by Nakano et al. (2003a) by a replacement of the last two amino acids by two aspartate residues of the *C*-terminus of Spx (a *ssrA*-like tag was altered and rendered the Spx more stable). In turn, the detected half-life was increased at least five times (Figure 4). Nevertheless, the Strep-tagged MgsR version is still functionally active and able to induce target gene expression of the marker gene *ydbD* as described before (Figure 5) (Reder et al., 2008). MgsR breakdown and regulatory effects of R/E-substitutions with previously identified arginine phospho-sites (Trentini et al., 2016) can be precisely determined with the MgsR^C–Strep^ fusion protein. Finally, a model system was generated to follow MgsR activity as well as MgsR stability simultaneously.

### MgsR Destabilization by Putative Arginine Phospho-Sites

McsB-dependent arginine phospho-sites were identified by different approaches (Fuhrmann et al., 2009; Elsholz et al., 2012; Schmidt et al., 2013; Trentini et al., 2014, 2016). In this context, phosphorylations of arginine residues R17 and R95 were described for MgsR (Trentini et al., 2016).

The adjacent sequences of R17 (region of CxxC motif) and R95 (RRPI motif) are conserved between MgsR and Spx (here R14 and R92) as well as in the *Bacillus* MgsR protein family (Supplementary Figure S2C). A structure of oxidized Spx (Newberry et al., 2005) demonstrates that both R14 and R92 are immediately located behind the redox-sensitive CxxC motif (Supplementary Figures S2A,B). Therefore, a phosphorylation could influence the regulatory protein activity by changing the positive charge of arginine residue and in turn the spatial localization of redox sensitive cysteine residues.

To investigate the impact of arg-P, every single arginine of MgsR was substituted by glutamate (R-E), whereas R94 and R95, were changed together to avoid an intramolecular arg-P switch from R94 to R95 or *vice versa* (Figure 4). Thus, a complex overview of the arg-P impact for MgsR was generated. Taken together, three “impact-clusters” were identified. The greatest effect was detectable for R33E and R94/95E (ochre bars). These rapid degradations were comparable with a full R/E mutant of MgsR^C–Strep^ in which all arginine residues were substituted by glutamate (gray bar). As anticipated, an opposed effect causing a stabilization (half-life ca. 23.2 min) was observed for MgsR R94/95K mimicking the permanently positively charged but non-phosphorylatable form, strongly indicating that these residues are decisive for targeting and turnover of MgsR (Supplementary Figure S4).

In contrast, the point mutation R63E (red bar) showed no reduction on half-life indicating that MgsR phosphorylation is not a randomized process and distinct phospho-sites are important for targeted protein degradation, especially for MgsR. Beyond that, point mutations R17E, R37E and R42E (blue bars) destabilize MgsR^C–Strep^ approximately by half, causing only a moderate effect.

Especially the merely moderate effect of R17E was surprising due to its close proximity to the redox-sensitive CxxC motif. Nevertheless, the arg-P mimic in this region seems to play only a marginal role for MgsR stability. Actually, it was supposed that even small conformational changes especially in the immediate proximity of functional domains, i.e., the redox-sensitive domain, may result in an enhanced recognition and degradation by the Clp proteases. Taken together, it was demonstrated that an R/E mediated phospho-mimicry exhibited different effects on MgsR and that the destabilization depends on specific arginine residues.

### Arginine Phosphorylation Mimicry and Impact on MgsR Activity

Based on the accepted assumption that phosphorylation events can activate or inactivate protein functions directly (Mijakovic et al., 2005, 2016; Macek et al., 2019), the activity of the MgsR as a regulator was examined in parallel. For this purpose, fluorescence-based Western blot analyses were conducted to follow the expression of the MgsR-inducible target gene *ydbD* (Reder et al., 2008).

Initially, *ydbD* induction of *B. subtilis* wild type 168 (positive control) was compared with the xylose induced *mgsR*^WT C–Strep^ version as well as its isogenic Δ*mgsR* deletion mutant (negative control). In the deletion mutant *ydbD* expression depends only on the remaining low level activity of the SigB-type promoter (Reder et al., 2008). A rather similar YdbD induction kinetics between wild type and xylose induced MgsR^WT C–Strep^ revealed that the Strep-tag interferes only slightly but recognizable with MgsR activity (Figure 5A).

In the next step, comparisons of MgsR^WT C–Strep^ and the Δ*mgsR* deletion mutant with all R/E point mutants were conducted to distinguish between MgsR-specific induction of the *ydbD* gene expression and the mentioned SigB-mediated background level (Figure 5A).

Subsequently, it was found that all but one point mutant (R42E) more or less lost their functional activity to induce *ydbD* expression in comparison to the wild-type MgsR suggesting that the positively charged arginine residues may also be essential for DNA or RNA polymerase α-CTD binding of MgsR (Figure 5B, Supplementary Figure S4).

MgsR R42E was not inhibited in its activity, which does not go along with the fast degradation, whereas the stable R63E showed no detectable transcriptional activity. These data further substantiate the idea that PTMs could influence a target protein with different outcomes.

## Discussion

The fundamental principles of MgsR proteolysis are quite well understood. Nevertheless, detailed regulatory and fine-tuned processes for Clp-dependent MgsR degradation are still not known. To gain deeper insights, an *in vivo* interaction analysis called SPINE, was utilized to seize and to identify the relevant interaction partners of MgsR during stress (Figure 1). Apart from anticipated interacting proteins such as ClpX and ClpC for MgsR degradation (Reder et al., 2012) or RpoA, the α subunit of RNA polymerase, where MgsR and Spx acts as transcriptional gene regulators (Nakano et al., 2003a, b; Newberry et al., 2005; Reder et al., 2012) directing the polymerase to specific σ^B^-dependent promoters, also new candidates were uncovered. All in all, 44 candidates including the arginine kinase McsB were identified (Figure 1).

To investigate the impact of McsB on MgsR degradation during secondarily induced oxidative stress, the MgsR proteolysis was determined by radiolabeling and immunoprecipitation (Reder et al., 2012) in a Δ*mcsB* deletion mutant with the result of a significant increase of MgsR half-life (Figure 2). This observation seems to be in accordance with the hypothesis that arg-P marks proteins for degradation in analogy to eukaryotic ubiquitin-labeling (Trentini et al., 2016).

McsB itself possesses an auto-kinase, which can be remarkably enhanced by the double-zinc-finger McsA partner protein, where the second zinc finger mediates McsA-McsB interaction (Kirstein et al., 2005; Elsholz et al., 2011b). All crucial arginine residues for the kinase and stimulated kinase reaction of McsB were identified in McsB as well as McsA by a radioactive *in vitro* phosphorylation approach with numerous R-K exchanges in McsB as well as McsA (Figure 3, Supplementary Figure S1). The results of these experiments fit perfectly with the recently published 3D structure of McsB and, moreover, identified for the first time, which arginine residues are generally needed for the McsB kinase activity, which are dispensable or required for a McsA-stimulated kinase reaction.

Surprisingly, the influence of an arginine kinase inactive McsB version (*mcsB*^C167S^) on MgsR proteolysis was not as strong as Δ*mcsB* suggesting that McsB^C167S^ is not able to activate itself, the ATPase ClpC by arg-P (Kirstein et al., 2005; Elsholz et al., 2012) and to phosphorylate MgsR, whereas it still functions as adaptor protein for degradation. The difference between Δ*mcsB* and *mcsB*^C167S^ point to the effect of the kinase-independent adaptor protein function of McsB for MgsR degradation (Figure 2).

Furthermore, a comparison of the MgsR stability in Δ*clpX* and Δ*clpC* mutants exhibits that the impact of McsB appeared similar to ClpX suggesting that McsB operates as an adaptor for ClpX during MgsR degradation (Figure 2). However, until now there is no final experimental proof that McsB acts as ClpX adaptor (for example by *in vitro* degradation experiments). Therefore, it remains an open question whether MgsR is directly or indirectly recognized and targeted for ClpXP degradation. Nevertheless, it is interesting to note that the *N*-terminal domain of ClpX is homologous to ClpE, which is usually involved in adaptor protein interaction. Strikingly, ClpE was also identified as interaction partner of McsB (Elsholz et al., 2011a) and would substantiate the prediction of a direct interaction of McsB as adaptor for ClpX.

Subsequently, phoshomimetic MgsR mutants were envisioned to investigate the role of specific arg-P’s for ClpXP degradation and protein activity. Therefore, the influence of arg-P on every single MgsR arginine residue was analyzed with regard to proteolysis and transcriptional activity, despite the fact, that in the present experimental setup (*mcsB* + cells) additional artificial negative charges might alter MgsR stability and/or functionality. For this purpose, an expression system was constructed and integrated into the non-essential *amyE* site, in which *mgsR* gene expression only depends on xylose addition (Supplementary Figure S3). Positively charged arginine residues (R) were substituted by negatively charged glutamate (E) resulting in a so-called phospho-mimicry (R/E). Moreover, the addition of a *C*-terminal Strep-tag to MgsR increased its half-life approximately fivefold, which allowed it to analyze the influence of every R/E exchange more precisely (Figure 4). Based on these studies, substitution mutants R33E and R94/95E exhibit the most significant destabilizing effects on MgsR, whereas R17, R37, R42, and R63 show only a minor or no effect. Accordingly, it is postulated that arginine residues R33 and R94/95 are the decisive MgsR arginine residues mediating a faster McsB-dependent degradation of MgsR. The half-life of MgsR R94/95K mimicking the permanently positively charged and non-phosphorylatable form was extended to 23.2 min indicating that these residues are decisive for targeting and turnover (Supplementary Figure S4). Although, very recently we found that MgsR^R94/95E^ was nearly completely stabilized in a *mcsB* deletion mutant showing that the phosphomimetic residues (R94/95E) alone are not sufficient for ClpXP-dependent degradation and that McsB, perhaps as adaptor, is absolutely required for proteolysis (unpublished observations). An increased degradation of the MgsR paralog Spx was also recently described in an *ywlE* (arginine phosphatase) mutant of *B. subtilis*, suggesting that McsB-dependent arg-P stimulates Spx proteolysis, here however, in cells lacking ClpX (Rojas-Tapias and Helmann, 2019). However, in doing these studies it should be kept in mind, that single amino acid replacements can also cause unusual or altered protein conformation impacting the stability independently from the Clp protease complexes. Moreover, not all Gram-positive bacteria possess the McsB and McsA proteins (for example, Lactobacillales) and can probably not use arg-P as a PTM system for a fine-tuned Clp protein degradation.

Finally, the transcriptional activity of MgsR was monitored by the induction of the MgsR-dependent *ydbD* gene on the protein level by Western-blots using an YdbD antibody (Reder et al., 2008). A comparison between wild type MgsR, all R/E substituted MgsR point mutants and MgsR R94/95K revealed that all but one (R42E) phospho-mimicries inactivated MgsR as transcriptional activator, however to different extent. Likewise, a reduction of the Spx activity was recently observed for Spx^R14K^, Spx^R92K^, and Spx^R112K^ and generally in a *ywlE* (arginine phosphatase) mutant with regard to the Spx-dependent *trxB* transcription (Rojas-Tapias and Helmann, 2019). Former studies demonstrated a strong arginine-DNA affinity and it is therefore not surprising that many R/E modifications reduce the ability of MgsR to direct the RNA polymerase to their specific promoters (Luscombe et al., 2001). As a consequence it is suggested that arg-P events have the predominantly purpose to inactivate MgsR resulting in a specific down-regulation of the SigB dependent general stress response.

Furthermore, the special cases of R63E and R42E demonstrate that a mimicked protein phosphorylation can have opposite effects with regard to activity and degradation. The MgsR R63E point mutant is comparably stable as the wild type protein, but possesses almost no activity. In contrast, MgsR R42E was rather quickly degraded but remained completely active. These observations underline that the effect of an arg-P highly depends on the location of the specific arginine residue.

In conclusion, regulation of MgsR activity is caused by a complex interplay of arg-P’s, which directly influence the inactivation and degradation of MgsR and, on top of that, McsB autokinase activity and McsA stimulation modulate the functionality of the McsB adaptor.

## Materials and Methods

### Bacterial Strains and Conditions of Cultivation

All strains for experiments were listed in Table 1. The strains were cultivated in a synthetic medium (Stülke et al., 1993) containing 15 mM (NH_4_)_2_SO_4_, 8 mM MgSO_4_.7 H_2_O, 27 mM KCl, 7 mM Na_3_ citrate.2 H_2_O, 50 mM Tris, 0.6 mM KH_2_PO_4_, 2 mM CaCl_2_.2 H_2_O, 1 μM FeSO_4_.7 H_2_O, 10 μM MnSO_4_.4 H_2_O, 0.2% (w/v) glucose, 0.78 mM *L*-tryptophan, and 4.5 mM *L*-glutamate. To allow growth of the *clp* mutant strains, 0.01% (w/v) yeast extract had to be added to the minimal medium. Cultivation was initiated by obtaining a starting OD_500_ of 0.05 with exponential growing cells in pre-warmed medium. In general, 100 ml cultures were grown in 500 ml Erlenmeyer flasks at 180 rpm and 37°C.

**TABLE 1:** List of used *B. subtilis* strains.

**Strain**	**Relevant features**	**References**
168	*trpC2*	Burkholder and Giles (1947)
BAR 36	*trpC2*Δ*mgsR*:*erm*; *amyE*: +200bp-*mgsR*^C–Strep^ (*cat*)	Reder et al. (2012)
BEK 89	*trpC2 lys-3*Δ*mcsB*:*aphA3*	Krüger et al. (2001)
BAE 74	*trpC2 mcsB*^C167S^ (*spc*)	Kirstein et al. (2005)
QPB418	*trpC2* Δ*clpC*:*tet*	Pan et al. (2001)
BUG 6	*trpC2* Δ*clpX*:*aphA3*	Kock et al. (2004)
BLL33	*trpC2* Δ*clpX*:*erm*	This study
BEK 90	*trpC2*Δ*clpX*:*aphA3*	Gerth et al. (2004)
BAR1	*trpC2*Δ*mgsR*:*erm*	Reder et al. (2008)
BAE 87	*trpC2 clpC*^R254K^ (*spc*)	Elsholz et al. (2012)
BFT 11	*trpC2 clpC*^R254K^ (*spc*); Δ*clpX*:*aphA3*	This study
BSAM 16	*trpC2*Δ*mgsR*:*erm*; *amyE*: +34bp-*mgsR*^WT^ (*cat*)	This study
BLL34	*trpC2*Δ*mgsR*:*erm*; *amyE*: +34bp-*mgsR*^WT C–Strep^ (*cat*)	This study
BLL35	*trpC2*Δ*mgsR*:*erm*; *amyE*: +34bp-*mgsR*^R17E C–Strep^ (*cat*)	This study
BLL36	*trpC2*Δ*mgsR*:*erm*; *amyE*: +34bp-*mgsR*^R94/95E C–Strep^ (*cat*)	This study
BLL50	*trpC2*Δ*mgsR*:*erm*; *amyE*: +34bp-*mgsR*^R33E C–Strep^ (*cat*)	This study
BLL51	*trpC2*Δ*mgsR*:*erm*; *amyE*: +34bp-*mgsR*^R37E C–Strep^ (*cat*)	This study
BLL52	*trpC2*Δ*mgsR*:*erm*; *amyE*: +34bp-*mgsR*^R42E C–Strep^ (*cat*)	This study
BLL53	*trpC2*Δ*mgsR*:*erm*; *amyE*: +34bp-*mgsR*^R63E C–Strep^ (*cat*)	This study
BLL54	*trpC2*Δ*mgsR*:*erm*; *amyE*: +34bp-*mgsR*^R17/33/37/42/63/94/95E C–Strep^ (*cat*)	This study
BLL55	*trpC2*Δ*mgsR*:*erm*; *amyE*: +34bp-*mgsR*^R94/95K C–Strep^ (*cat*)	This study

## Construction of Mutants

All primers for the construction of *in vivo* mutant strains were listed in Supplementary Table S2. Deletion or point mutations were cloned by using linear DNA fragments constructed by the principle of LFH-PCR (Wach, 1996). Purification and fusion of PCR products were conducted as described previously (Reder et al., 2008). All chromosomal changes were selected by the application of resistance markers that were flanked by homologous up- and downstream sequences of the respective gene. Mutants were selected on LB agar plates containing either erythromycin (5 μg ml^–1^) plus lincomycin (25 μg ml^–1^), chloramphenicol (5 μg ml^–1^), kanamycin (10 μg ml^–1^), spectinomycin (200 μg ml^–1^), tetracycline (17 μg ml^–1^), or a combination of them (final concentration).

The corresponding chromosomal mutant DNA was checked by sequencing with respect of correctness of resistance marker or point mutation and to exclude further undesirable base substitutions in the up- or downstream area.

### *In vivo* Formaldehyde Crosslinking

When cultures reached an optical density OD_500_ 0.4, a formaldehyde solution was added to a final concentration of 0.6% as described previously (Herzberg et al., 2007). After the addition of formaldehyde, the cultures were incubated for 20 min at 37°C, cooled down on ice, harvested by centrifugation and washed once with ice cold buffer W (200 mM NaCl, 50 mM Tris/HCl, and pH 8.0). Washed pellets were dissolved in 10 ml buffer W, cells were disrupted by a French press and protein lysates were separated from cell debris by centrifugation prior Strep-tag purification. A total of 30 μl aliquots of the purification steps were mixed with Laemmli buffer and were either directly subjected to SDS-PAGE or boiled for 20 min in Laemmli buffer prior to electrophoresis to reverse the formaldehyde cross-links. Gels were analyzed by silver staining and Western blot analysis with MgsR specific antibody. Whole lanes of Coomassie stained gels were cut in three or four pieces respectively and subjected to tryptic in gel digestion and subsequent analysis by mass spectrometry.

### Data Evaluation of the MgsR SPINE Assay

Proteins were identified by searching against a *B. subtilis* target-decoy protein sequence database (8294 entries) using Sorcerer^TM^-SEQUEST^®^ (Sequest version 2.7 rev. 11, Thermo Electron including Scaffold_3_00_02, Proteome Software Inc., Portland, OR, United States). The target-decoy database includes the complete proteome set of *B. subtilis* 168 (4,105 database entries) that was extracted from UniprotKB version 1.2.0.18 and an appended set of 4,147 reversed sequences and 42 sequences of common laboratory contaminants created by BioworksBrowser version 3.2 (Thermo Electron Corp.) (Elias and Gygi, 2007). A Sequest search was carried out considering the following parameter: a parent ion mass tolerance 10 ppm, fragment ion mass tolerance of 1.0 Da. Two missed tryptic cleavages were allowed. Methionine oxidation (+15.994915 Da) and cysteine carbamidomethylation (+57.021464 Da) were set as variable modifications. Proteins were identified by at least five peptides applying a stringent SEQUEST filter. Sequest identifications required at least Cn scores of greater than 0.1 and XCorr scores of greater than 1.9, 2.2, 3.3, and 3.75 for singly, doubly, triply and quadruply charged peptides.

### Stress Exposure and Cell Sampling

At OD_500_ of 0.4, unstressed cells were sampled as a control before pX based gene expression was initialized by 0.3% (w/v) xylose or secondary oxidative stress induced by 4% (v/v) ethanol. All samples were immediately chilled down with liquid nitrogen and pelleted/spun down by centrifugation at 10,000 *g* and 4°C for 3 min. Storage of samples for next preparations was ensured at −70°C.

### Radioimmunoprecipitation, Signal Quantification and Half-Life Determination

Cultivation and following experimental procedures were performed as described previously (Gerth et al., 2008) with modifications. Cells were grown in synthetic Belitsky minimal medium, which was supplemented with 0.01% yeast extract to ensure the growth of the *clp* mutants. Pulse-labeling was initiated with *L*-^35^*S*-methionine at OD_500_ of 0.4 as described before (Gerth et al., 2008). The *mgsR* expression was induced by addition of 0.3% (w/v) xylose after 2 min of labeling. Furthermore, MgsR activation was accomplished by 4% (v/v) ethanol stress after 2and 10 min later incorporation of radioactive methionine was stopped with 10 mM *L*-^32^*S*-methionine. At this time point, a control sample was taken followed by a time kinetic after 10, 20, 30, 40, 50, and 120 min. Collected cells were disrupted in lysis buffer [50 mM Tris/HCl, pH 7.5, 5 mM EDTA, 4 mg ml^–1^ lysozyme, protease inhibitor (complete Mini)] for 20 min at 42°C and addition of 2% (w/v) SDS.

After removing the cell debris by centrifugation (30 min, 10,000 *g*, and 4°C), a purified MgsR-specific antibody was added to the protein extract and was incubated overnight at 4°C. The MgsR-antibody and the MgsR protein were recovered by utilization of protein G-coated Dynabeads (Thermo Fisher Scientific). Samples were subjected to SDS-PAGE (15%) and gels were dried on Whatman paper using a heated vacuum dryer. Gels were exposed to storage phosphor screens (Molecular Dynamics) for a time span ensuring the utilization of the whole dynamic range that will be determined by the strongest signal on the gel. The strongest signal must not exceed the maximum intensity level of 100,000 and must not be below a level of 95,000. Therefore, the screens were scanned using a Typhoon 9400 scanner at a resolution of 200 nm and a color depth of 16bits (65,536 gray levels) and the exposition times must be monitored and adapted to meet the criteria mentioned above. The quantification of the signals was performed with the ImageQuant TL Software from GE Healthcare Life Sciences or the freely available ImageJ software (Rasband, W.S., ImageJ, National Institutes of Health, Bethesda, MD, United States). All experiments were carried out in triplicate. The quantification of the MgsR-specific signals was performed using the ImageJ software version 1.43u. Mean values and standard deviations were calculated and all values were normalized to the initial value of the time-series (*t*_0_ min = 100%). A non-linear regression model (non-linear least squares) was used to obtain the best fitting curve function on the logarithmic values [equation A: *y* = 2 − *a* (1 − e^(–^*^x^*^/^*^b^*^)^)]. To determine the half-life (*t*_1__/__2_) the equation was solved for *x* (time) in *y* = 50 (50% intensity) [equation B: (*x*) = ln (1 − log_10_50 − log_10_100/(−a)) × (−b)] (Reder et al., 2018).

### Fluorescence Based Western Blot Analysis

Cell samples were disrupted by ultrasonic treatment as described before (Gerth et al., 2008) and protein amount was determined by NanoDrop^®^ ND-1000 Spectrophotometer. Protein samples were subjected by SDS-PAGE (8-16% Mini-PROTEAN^®^ TGX Stain-Free^TM^ Protein Gels, Biorad). A total of 50 μg soluble protein was separated in each gel lane. After blotting, the blocked membrane was incubated with the primary antibody MgsR antibody (1:5,000) and YdbD antibody (1:5,000) in Odyssey^®^ blocking buffer (TBS) plus 0.2% (v/v) Tween-20 overnight at 4°C. Next day, the membrane was washed four times in TBS buffer (50 mM Tris, 150 mM NaCl, and pH 7.6) plus 0.1% Tween-20. After an incubation of the membrane with the secondary antibody solution (IR Dye^®^ 800CW Goat anti-Rabbit (1:15,000) plus 0.2% (v/v) Tween-20 plus 0.01% (w/v) SDS in Odyssey^®^ blocking buffer (TBS) for 1 h, a further wash procedure was performed in TBS buffer plus 0.1% Tween-20 for five times. The corresponding protein signals on dried membrane were scanned by Odyssey^®^ CLx Imaging System (LI-COR Biosciences).

### *In vitro* Phosphorylation Assay

His_6_-tagged McsB and McsA, which were cloned into the pRSETA vector (Invitrogen) as *N*-terminal His_6_ variants, were expressed in *Escherichia coli* BL21 DE3 pLysS and natively purified by imidazole elution from Ni-NTA agarose columns according to standard protocols. Site-directed mutagenesis of all R-K exchanges was performed with pRSETA-*mcsB* and pRSETA-*mcsA* (Krüger et al., 2001) as template and the respective primer (Supplementary Table S1) using the Gene-Tailor-System or Gene-Art-System (Life Technologies). McsB and McsA were tested for phosphorylation by incubation at a concentration of 10 μM in phosphorylation assay buffer (25 mM Tris–HCl, pH 8, 300 mM NaCl, 5 mM MgCl_2_, and 1 mM DTT) at 30°C in the presence of 10 μCi of [γ-^32^P] ATP in a final volume of 15 μl. If not stated otherwise, samples of 10 μl were withdrawn after 20 min and mixed with 4 μl of 4× SDS sample buffer and resolved by 12% SDS-PAGE. Phosphorylation signals were detected by autoradiography with a Typhoon-Scanner (GE Healthcare) in phosphor-modus (633 nm, 390 BP, 100 *phosphor*, PMT 750V, 200 microns, *focal plane* +3 mm).

## Data Availability Statement

The datasets generated for this study are available on request to the corresponding author.

## Author Contributions

AR, UV, MH, and UG designed the work and interpreted the data. LL, AR, FT, FM, and JG performed the experiments, generated and interpreted the data. DB conducted the mass spectrometry and analyzed the data. UG, AR, LL, UV, MH, and KR analyzed the data and wrote the manuscript.

## Conflict of Interest

The authors declare that the research was conducted in the absence of any commercial or financial relationships that could be construed as a potential conflict of interest.

## Supplementary Material

FIGURE S1 Alignment of related McsB proteins (Bs, *Bacillus subtilis*; Bl, *B. licheniformis*; Bp, *B. pumilus*; Ba, *B. anthracis*; Lm, *Listeria monocytogenes*; Cd, *Clostridioides difficile*; Sa, *Staphylococcus aureus*). The catalytic active cysteines are labeled red, the conserved and exchanged arginines blue. The ATP:guanidino phosphotransferase (AP) domain (residues 1–263) was labeled yellow, the lid region light green (residues 210–220), the pArg-binding pocket light blue (residues 337–342) and the *C*-terminal dimerization domain (DD) ochre (residues from 264) according to Suskiewicz et al. (2019).

FIGURE S2 Structural and spatial organization of arginine residues in MgsR and Spx. **(A)** The structure of oxidized Spx (Newberry et al., 2005) depicts the conserved cysteine residues of the redox center in red (C10 and C13 for Spx and C13 and C16 for MgsR; CxxC motif) and arginine residues in the immediate vicinity in cyan (R14 and R92 for Spx; R17 and R95 for MgsR). **(B)** The surface structure of Spx (Newberry et al., 2005) was used to color conserved MgsR arginine residues cyan and the redox-sensitive CxxC-motif red. **(C)** A multiple sequence alignment of MgsR proteins of different *Bacillus* species and a comparison with the Spx paralog (*Bacillus subtilis* 168). The cyan color highlights the conservation of arginines in Spx and MgsR.

FIGURE S3 Schematic illustration of the pX-based xylose inducible system for *mgsR* expression.

FIGURE S4 Comparison of stability and activity for R94/95E and R94/95K substituted MgsR point mutants. **(A)** Radioimmunoprecipitation was performed for R94/95E and R94/95K substituted MgsR isoforms to determine the half-life of these two protein species. MgsR synthesis was induced by 0.3% (v/v) xylose and stress was provoked by 4% (v/v) ethanol. Samples were taken immediately after chasing reaction by ^32^*S*-methionine (0′) and 10, 20, 30, 40, 50, and 120 min later. **(B)** Fluorescence based Western blot analyses using YdbD and MgsR specific antibodies were conducted to follow the YdbD induction kinetic after the start of MgsR synthesis provoked by 0.3% (v/v) xylose and stress initiation by 4% (v/v) ethanol. Samples were taken 10 and 20 min after stress exposure, whereas a control (co or 0′) was sampled immediately before. In this way, activities of Strep-tagged wild-type MgsR (positive control) was compared with corresponding R94/95E and R94/95K point mutants, whereas Δ*mgsR* served as negative control. (C) All detected MgsR (blue) and YdbD (orange) signals of the Western blot were illustrated in a column chart.

TABLE S1 Primers used for *mcsB*/*mcsA* mutagenesis and *in vitro* phosphorylation.

TABLE S2 List of used primers for MgsR mutagenesis.
