# Supplementary material for: The Involvement of the McsB Arginine Kinase in Clp-Dependent Degradation of the MgsR Regulator in Bacillus subtilis
Source: Front Microbiol. 2020 May 12;11:900. doi: 10.3389/fmicb.2020.00900 (PMC7235348; doi:10.3389/fmicb.2020.00900)
Supplement: FIGURE S1 — Alignment of related McsB proteins (Bs, Bacillus subtilis; Bl, B. licheniformis; Bp, B. pumilus; Ba, B. anthracis; Lm, Listeria monocytogenes; Cd, Clostridioides difficile; Sa, Staphylococcus aureus). The catalytic active cysteines are labeled red, the conserved and exchanged arginines blue. The ATP:guanidino phosphotransferase (AP) domain (residues 1–263) was labeled yellow, the lid region light green (residues 210–220), the pArg-binding pocket light blue (residues 337–342) and the C-terminal dimerization domain (DD) ochre (residues from 264) according to Suskiewicz et al. (2019). [file Image_1.pdf]

Supplementary Figure S1

```
Bs             1 MSLKHFIQDALSSWMKQKGPESDIVLSSRIRLARNFEHIRFPTRYSNEEASSIIQQFEDQ
Bl             1 MSLQHFIQNALSNWMRQEGPESDIVLSSRIRLARNLDKVRFPTLFSNEEASAIIALFEEQ
Bp             1 MSLQHFIQDALSQWMKQKGPESDIVLSSRIRLARNLEHVRFPTQFSQAEAEAVLQQFEQK
Ba             1 MSLDKIMNEAISPWMKGDGPDSDIVLSSRIRLARNFKKYQFSTMQNEEETKQIQELFKKE
Lm             1    MNVFEPRLSSWLENAGDDDDVVLSSRIRLARNLKDEQFPVYEQKEEIVDNIAEV---
Cd             1                    MKENIVMKSRVRLARNLNNYPFPNKLDKECAMEIIEKVKNA
Sa             1  MMTHNIHDNISQWMKSN-EETPIVMSSRIRLARNLENHVHPLMYATENDGFRVIN----
consensus      1                         V  SR RLARN

Bs            61 FSEQEIPGIGKFVLIRMNDAQPLEKRVLVEKHLISPNLT-ESPFGGCLLSENEEVSVMLN
Bl            61 FTGYEVPGIGKFELVKMDQVQPLEKRVLVEKHLISPHLT-EASFGACLLSENEEVSIMLN
Bp            61 FASQEVKDIGNFVLIRMNETQPLAKRVLVEKHLISPNLA-ESRFGGCLLSENEEISVMLN
Ba            61 FINKTVEPFGEFELLKMNELTPLQRRVLVEKHLISPNLA-GTEYGACLLSESEHISVMLN
Lm            55 -------FDDNFTLIKMNQISLLQKALLVEKHLISPYMMNKSEYGAVLLNEEENVSIMLN
Cd            42 FINSNLEQKEEFDFYKIEDLDQSKKMLMVEEHIISPDLA-ENDKSAVIVKKDKTISIMIN
Sa            55 ---EVQDALPNFELMRLDQMDQQSKMKMVAKHLISPELI-KQPAAAVLVNDDESLSVMIN
consensus     61            F                V  H ISP                   S M N

Bs           120 EEDHIRIQCLFPGFQLLEAMKAANQVDDWIEEKVDYAFNEQRGYLTSCPTNVGTGLRASV
Bl           120 EEDHIRIQCLFPGFQLSEALKAANQVDDWIEDRIDYAFSEQRGYLTSCPTNVGTGLRASV
Bp           120 EEDHIRIQCLFPGFQLANALKAANQVDDWIEEQVDYAFSEKRGYLTSCPTNVGTGIRASV
Ba           120 EEDHIRIQCLFSGLQLSEALQSANQIDNWIEKEVEYAFDESLGYITSCPTNVGTGLRASV
Lm           108 EEDHLRIQCMTPGLRLFDALEAALQIDGYVEEKLSYAFDKEFGYLTSCVTNIGTGMRASV
Cd           101 EEDHIRIQTICDDLNLEYAYSVANEIDDLLESSLEYAFNTKLGYLTSCPTNTGTGMRASV
Sa           111 EEDHIRIQAMGTDTTLQALYNQASSIDDELDRSLDISYDEQLGYLTTCPTNIGTGMRASV
consensus    121 EEDH RIQ       L      A   D               GY T C TN GTG RASV

Bs           180 MMHLPALVLTRQINRIIPAINQLGLVVRGIYGEGSEAVGNIFQISNQITLGKSEQDIVED
Bl           180 MMHLPALVLTRQINRIIPAINQLGLVVRGIYGEGSEALGNIFQISNQITLGKSEHDIVED
Bp           180 MMHLPALALTRQMNRIIPAINQLGLVVRGIYGEGSEAIGNIFQISNQMTLGQSEEDIVDD
Ba           180 MIHLPGLVLTKRISRIIQVIQKLGLVVRGIYGEGSEALGNIFQVSNQMTLGKSEEDIIAD
Lm           168 MVHLPGLVTTKRIKSVIEAIRSLGFVVRGIYGEGSMPASNIFQVSNQVTLGKTETEIVED
Cd           161 MMHLPALSQLGYMDELYKISSQIGIAIRGIYGERTEALGNIYQISNQLTLGRTESNIIEN
Sa           171 MLHLPGLSIMKRMTRIAQTINRFGYTIRGIYGEGSQVYGHTYQVSNQLTLGKSELEIIET
consensus    181 M HLP L                G   RGIYGE         Q SNQ TLG  E  I

Bs           240 LNSVAAQLIEQERSAREAIYQTSKIELEDRVYRSYGVLSNCRMIESKETAKCLSDVRLGI
Bl           240 LNSVVAQLIEQERSARKALYQTSQIELEDRVYRSYGVLSNCRMIESKETARCLSDVRLGI
Bp           240 LNSVTAQLIEQERSARKALYQTSKIELEDRVYRSLGILANCRMIESKETAKCLSDVRLGI
Ba           240 LKSVIQQIIQQEKMARELIVQNSSIELEDKVYRSYGILANSRLIQSAEAANCLSDLRLGI
Lm           228 LTQVMEQIIMQERVARTTLKQKFHIALEDRVFRSYGLLMNCRIISMKEASDAISDIRLGV
Cd           221 VSGLTKDAISKEIKAREILQKKLGIKLEDKIFRSIGTLENSRVMSSAEAMSHLSNIKMGI
Sa           231 LTEVVNQIIHEEKQIRQKLDTYNQLETQDRVFRSLGILQNCRMITMEEASYRLSEVKLGI
consensus    241         I  E   R            D   RS G L N R     E     S    G

Bs           300 DLGIIKGLSSNILNELMILTQPGFLQQYSGGALRPNERDIRRAALIRERLHLEMNGKRQE
Bl           300 DLGIIKGLSSNILNELMILTQPGFLQQYSGGALRPNERDIRRAALIRERLKLENNGNRQE
Bp           300 DLGIIKGLSSNILNELMILTQPGFLQQYSGGALEPNERDIKRAAIIRERLRLEMHRNGQE
Ba           300 DLGYIQGISRNILTELMVLTQPGILQQYAGGPLGPEERDYRRATLIRERLRIEKN-----
Lm           288 ELGFFEHISRQKMNELVLFSQPAFLRREAGRDMDELEEKVIRAKVIREILGDK-------
Cd           281 EMNYIDKLDLKAIEQLMIGIQPAHQSIMYK-SDDVENRDINRATYIRETLEKLRGGMNCE
Sa           291 DLNYIELQ-NFKFNELMVAIQSPFLLDEED----DKSVKEKRADILREHIK---------
consensus    301                L    Q                    RA   RE

Bs           360 DESI
Bl           360 DETI
Bp           360 DETI
Ba
Lm
Cd           340 LQ
```
