# Supplementary material for: The Involvement of the McsB Arginine Kinase in Clp-Dependent Degradation of the MgsR Regulator in Bacillus subtilis
Source: Front Microbiol. 2020 May 12;11:900. doi: 10.3389/fmicb.2020.00900 (PMC7235348; doi:10.3389/fmicb.2020.00900)
Supplement: FIGURE S2 — Structural and spatial organization of arginine residues in MgsR and Spx. (A) The structure of oxidized Spx (Newberry et al., 2005) depicts the conserved cysteine residues of the redox center in red (C10 and C13 for Spx and C13 and C16 for MgsR; CxxC motif) and arginine residues in the immediate vicinity in cyan (R14 and R92 for Spx; R17 and R95 for MgsR). (B) The surface structure of Spx (Newberry et al., 2005) was used to color conserved MgsR arginine residues cyan and the redox-sensitive CxxC-motif red. (C) A multiple sequence alignment of MgsR proteins of different Bacillus species and a comparison with the Spx paralog (Bacillus subtilis 168). The cyan color highlights the conservation of arginines in Spx and MgsR. [file Image_2.pdf]

Supplementary Figure S2

A

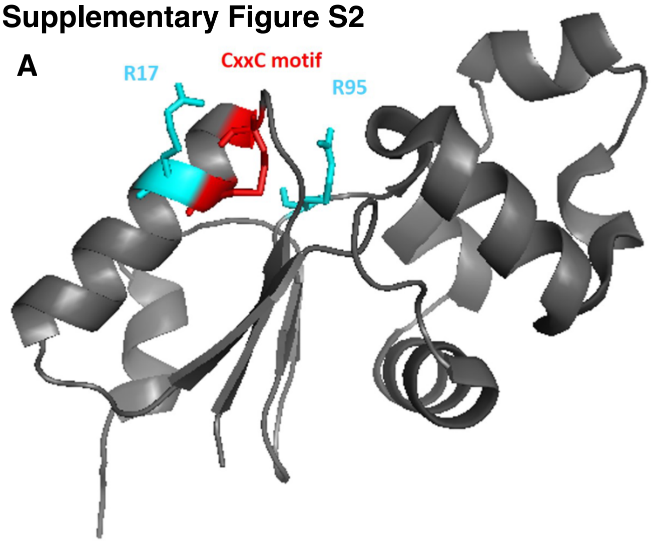

B

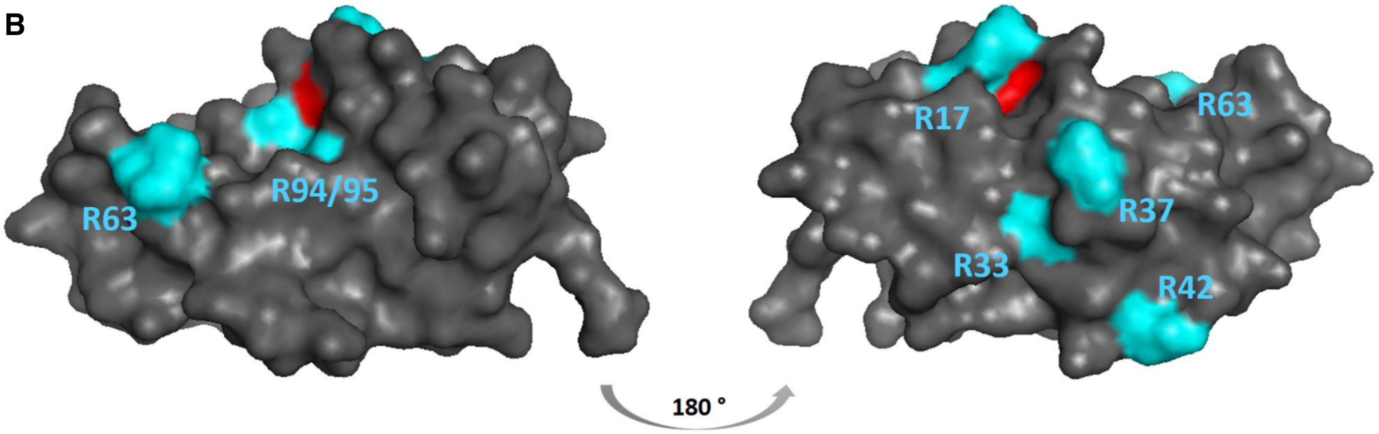

C

```
                                       R17                    R33  R37   R42
                                        ▼                      ▼    ▼     ▼
                         :*:*: ********:: **: *:* * **::* *  : :*:* ** :* :* ***:
   Spx                 ---MVTLYTSPSCTSCRKARAWLEEHEIPFVERNIFSEPLSIDEIKQILRMTEDGTDEII  57
   MgsR                MEQQLTFYSYPSCTSCRKTKHWLKAHQIEFNERHLFRETPTREELKYILSLTTEGIDEIL  60

B. subtilis            MEQQLTFYSYPSCTSCRKTKHWLKAHQIEFNERHLFRETPTREELKYILSLTTEGIDEIL  60
B. megaterium           MEEIKFYTYPSCTSCRKTKKWLVKEDVNFKERHLFRETPTHKEMLELLSMTTEGMDELL  59
B. licheniformis        MEELTFYSYPSCTSCRKTKHWLKANQVDFTERHLFRETPNQDELKHILSLTTEGIDEIL  59
B. pumilus              MSDITFYSYPSCTSCRKTKHWLKANQIDFKERHLFRETPTLEELKKILSLTTEGMDEIL  59
B. amyloliquefaciens    MKELIFYSYPSCTSCRKTKHWLKAHNIDFHERHLFRETPTIDELKQILSLTTEGIDEIL  59
B. velezensis           MKELIFYSYPSCTSCRKTKHWLKAHNVDFQERHLFRETPTIDELKQILSLTTEGIDEIL  59
B. atrophaeus           MEELTFYSYPSCTSCRKTKHWLKAHQIDFNERHLFRETPTMEELKYILSLTTEGIDEIL  59
                          .:: **:***********:** .:::* *********. .*: :**:****:**:*
consensus                      FY YPSCTSCRKTK WL     F ERHLFRETP   E    LS TTEG DE L

                         R63                          R94/95
                          ▼                             ▼▼
                        :***:.*::**:*:*.* :::: .*: *:* ******::*:*:* :*** .*: ::  :*
   Spx                  STRSKVFQKLNVNVESMPLQDLYRLINEHPGLLRRPIIIDEKRLQVGYNEDEIRRFLPRK 117
   MgsR                 ATRSQTFKNLNLNIEEMTVNEVLELLIEKPKLLRRPILVDNKKLVIGYNPGELLKLSKKK 120

B. subtilis             ATRSQTFKNLNLNIEEMTVNEVLELLIEKPKLLRRPILVDNKKLVIGYNPGELLKLSKKK 120
B. megaterium           AKRSQEYKKLNVDVDSMTLSEVVNLLIEHPRLLRRPILTDGKKLVVGYNESALKNLVKKK 119
B. licheniformis        ATRSQAFKELNLDIEELTVSEVLELLIQKPKLLRRPIIVNGDKLVVGYNPGELLKLKK-H 118
B. pumilus              ATRSQAFKSLNLNINDLKVNEVLQLLIEKPKLLRRPIIIDGNKLVVGYNPGELMKLSKKK 119
B. Amyloliquefaciens    ATRSQTFKNLNLNLEEMTVNEVLKLLTEKPKLLRRPILIDHKKLVIGYNPGELMKLTKKK 119
B. velezensis           ATRSQTFKNLNLNIEEMTVNEVLKLLTEKPKLLRRPILIDRNKLVIGYNPGELMKLTKKK 119
B. atrophaeus           ATRSQTFKELNLNIEEMTVNEVLDLLIEKPKLLRRPILVDNRKLVIGYNPGELLKLTKKK 119
                        *.*** :*.**::::.:.:.**:.** ::*:******: :  ***:*** . * :* * :
consensus                A RSQ  K LN         EV  LL   P LLRRPI     KLV GYN   L  L K


                         .   .
   Spx                   VRSFQLREAQRLAN      131
   MgsR                  TVHQSA              126

B. subtilis              TVHQSA              126
B. megaterium            TSLASLVV            127
B. licheniformis         TLHRSVS             125
B. pumilus               AIHQSVS             126
B. amyloliquefaciens     TVHQSVS             126
B. velezensis            TVHQSVS             126
B. atrophaeus            TVHQSVS             126
                         :   *
consensus                    S
```
