# Supplementary figures and images for: The Involvement of the McsB Arginine Kinase in Clp-Dependent Degradation of the MgsR Regulator in Bacillus subtilis
Source: Front Microbiol. 2020 May 12;11:900. doi: 10.3389/fmicb.2020.00900 (PMC7235348; doi:10.3389/fmicb.2020.00900)

# Supplementary Figure S3

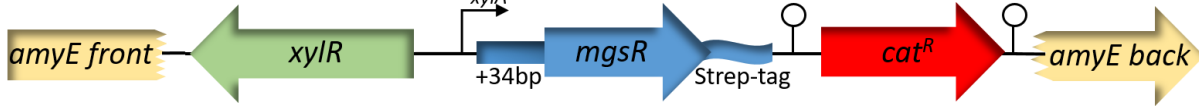

Supplement: FIGURE S3 — Schematic illustration of the pX-based xylose inducible system for mgsR expression. [file Image_3.pdf]

# Supplementary Figure S4

**A**

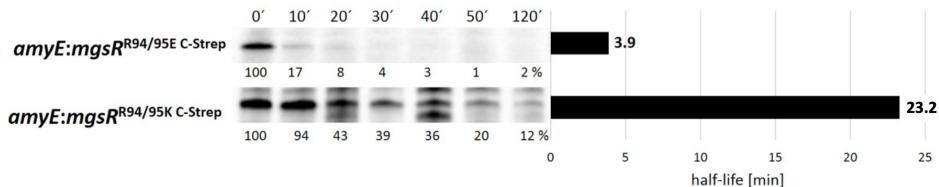

**B**

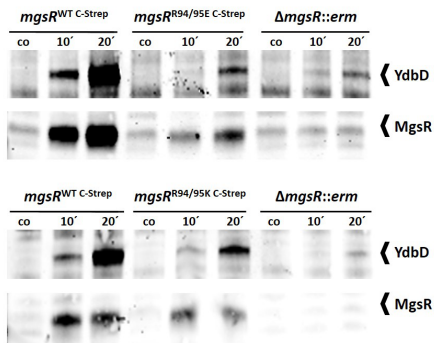

**C**

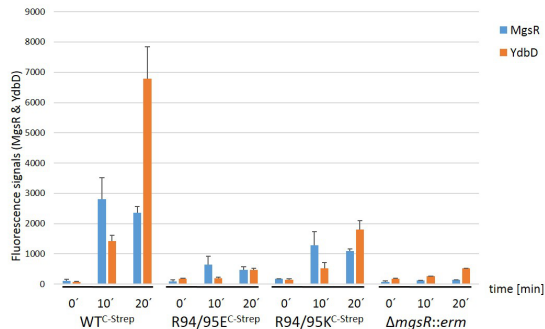

Supplement: FIGURE S4 — Comparison of stability and activity for R94/95E and R94/95K substituted MgsR point mutants. (A) Radioimmunoprecipitation was performed for R94/95E and R94/95K substituted MgsR isoforms to determine the half-life of these two protein species. MgsR synthesis was induced by 0.3% (v/v) xylose and stress was provoked by 4% (v/v) ethanol. Samples were taken immediately after chasing reaction by 32S-methionine (0′) and 10, 20, 30, 40, 50, and 120 min later. (B) Fluorescence based Western blot analyses using YdbD and MgsR specific antibodies were conducted to follow the YdbD induction kinetic after the start of MgsR synthesis provoked by 0.3% (v/v) xylose and stress initiation by 4% (v/v) ethanol. Samples were taken 10 and 20 min after stress exposure, whereas a control (co or 0′) was sampled immediately before. In this way, activities of Strep-tagged wild-type MgsR (positive control) was compared with corresponding R94/95E and R94/95K point mutants, whereas ΔmgsR served as negative control. (C) All detected MgsR (blue) and YdbD (orange) signals of the Western blot were illustrated in a column chart. [file Image_4.pdf]
