# Supplementary material for: The Involvement of the McsB Arginine Kinase in Clp-Dependent Degradation of the MgsR Regulator in Bacillus subtilis
Source: Front Microbiol. 2020 May 12;11:900. doi: 10.3389/fmicb.2020.00900 (PMC7235348; doi:10.3389/fmicb.2020.00900)
Supplement: TABLE S1 — Primers used for mcsB/mcsA mutagenesis and in vitro phosphorylation. [file Table_1.pdf]

Table S1: Primers used for *mcsB*/*mcsA* mutagenesis and *in vitro* phosphorylation

Primers for the construction of the plasmid pRSETA-*mcsB*

pRSETA-*mcsB*-for CGGGATCCATGTCGCTAAAGCATTTTATTCAGG

pRSETA-*mcsB*-rev GGGGTACCTCATATCGATTCATCCTCCTGTC

Primers for the construction of *mcsB* point mutants using pRSETA-*mcsB*

Bs-*mcsB*-R29K-for AGCAGCAAATACGTTTAGCAAGAAACTTTGAGCATA

Bs-*mcsB*-R29K-rev CTAAACGTATTTTGCTGCTGAGTACAATGTCACTTTC

Bs-*mcsB*-R31K-for CGCATAAAATTAGCAAGAAACTTTGAGCATATTCG

Bs-*mcsB*-R31K-rev TGCTAATTTTATGCGGCTGCTGAGTACAATGTCAC

Bs-*mcsB*-R34K-for TTAGCAAAAAACTTTGAGCATATTCGGTTCCCTACG

Bs-*mcsB*-R34K-rev AAAGTTTTTTGCTAAACGTATGCGGCTGCTGAGTAC

pRSETA_Bs168_*mcsB*_R86K_for
TTAGAAAAGAAGGTACTTGTTGAAAAACATCTAATCAGC

pRSETA_Bs168_*mcsB*_R86K_rev
AAGTACCTTCTTTTCTAAAGGCTGTGCATCATTCATTC

pRSETA_Bs168_*mcsB*_R125K_for
ACCATATTAAGATTCAGTGTCTTTTCCCTGGATTTCAGC

pRSETA_Bs168_*mcsB*_R125K_rev
CACTGAATCTTAATATGGTCCTCTTCATTTAGCATGACG

pRSETA_Bs168_*mcsB*_R161K_for
ATGAGCAAAAGGGATACTTAACCAGCTGTCCTACAAACG

pRSETA_Bs168_*mcsB*_R161K_rev
TAAGTATCCCTTTTGCTCATTGAATGCATAATCAACTTTTTC

Bs-*mcsB*-R176K-for GGTTTAAAAGCTTCGGTCATGATGCATCTGCCG

Bs-*mcsB*-R176K-rev CGAAGCTTTTAAACCAGTACCTACGTTTGTAGG

pRSETA_Bs168_*mcsB*_R190K_for
GGTTTTAACTAAGCAAATAAATCGAATTATACCGGCAATTAAT C

pRSETA_Bs168_*mcsB*_R190K_rev
ATTTATTTGCTTAGTTAAAACCAGCGCCGGCAGATGCATC

pRSETA_Bs168_*mcsB*_R194K_for
CAAATAAATAAGATTATACCGGCAATTAATCAATTAGGCTTAG

pRSETA_Bs168_*mcsB*_R194K_rev
CGGTATAATCTTATTTATTTGCCTAGTTAAAACCAGCGC

Bs-*mcsB*-R207K-for GTTGTTAAAGGAATTTATGGGGAAGGCAGCGAA

Bs-*mcsB*-R207K-rev AATTCCTTTAACAACTAAGCCTAATTGATTAATTG

pRSETA_Bs168_*mcsB*_R252K_for
GAACAAGAAAAGTCTGCCCGAGAAGCGATTTATCAAACTTC

pRSETA_Bs168_*mcsB*_R252K_rev
GGGCAGACTTTTCTTGTTCAATCAACTGAGCAGCAACAC

Bs-*mcsB*-R255K-for TCTGCCAAAGAAGCGATTTATCAAACTTCTAAAATC

Bs-*mcsB*-R255K-rev CGCTTCTTTGGCAGATCGTTCTTGTTCAATCAACTG

Bs-*mcsB*-R269K-for GAGGATAAAGTATATCGGTCATATGGGGTCTTGTCT

Bs-*mcsB*-R269K-rev ATATACTTTATCCTCAAGTTCGATTTTAGAAGTTTG

Bs-*mcsB*-R272K-for GTATATAAATCATATGGGGTCTTGTCTAATTGCCGG

Bs-*mcsB*-R272K-rev ATATGATTTATATACCCGATCCTCAAGTTCGATTTTAG

pRSETA_Bs168_*mcsB*_R281K_for
AATTGCAAGATGATAGAATCGAAAGAAACTGCAAAGTGC

pRSETA_Bs168_*mcsB*_R281K_rev
ATTCTATCATCTTGCAATTAGACAAGACCCCATATGACC

pRSETA_Bs168_*mcsB*_R296K_for
AGATGTTAAGTTAGGCATAGACTTAGGCATAATAAAAGG

pRSETA_Bs168_*mcsB*_R296K_rev
TGCCTAACTTAACATCTGAAAGGCACTTTGCAGTTTC

pRSETA_Bs168_*mcsB*_R333K_for

GAGGCGCTTTGAAGCCGAACGAAAGGACATTCGAAGAGC

pRSETA_Bs168_*mcsB*_R333K_rev

GTTCGGCTTCAAAGCGCCTCCCGAGTATTGCTGTAAAAAC

pRSETA_Bs168_*mcsB*_R337K_for

TCCGAACGAAAAGGACATTCGAAGAGCGGCTCTCATCAG

pRSETA_Bs168_*mcsB*_R337K_rev

GAATGTCCTTTTCGTTCGGTCGCAAAGCGCCTCCCGAG

Bs-*mcsB*-R341K-for ATTCGAAAAGCGGCTCTCATCAGAGAACGGCTTCAC

Bs-*mcsB*-R341K-rev AGCCGCTTTTCGAATGTCCCTTTCGTTCGGTCGC

Bs-*mcsB*-R346K-for CTCATCAAAGAACGGCTTCACTTAGAAATGAATGG

Bs-*mcsB*-R346K-rev CCGTTCTTTGATGAGAGCCGCTCTTCGAATGTCCC

pRSETA_Bs168_*mcsB*_R348K_for

CATCAGAGAAAAGCTTCACTTAGAAATGAATGGGAAAAGAC

pRSETA_Bs168_*mcsB*_R348K_rev

AGTGAAGCTTTTCTCTGATGAGAGCCGCTCTTCGAATG

pRSETA_Bs168_*mcsB*_R357K_for

AATGGGAAAAAGCAGGAGGATGAATCGATATGAGAATTCGG TACC

pRSETA_Bs168_*mcsB*_R357K_rev

ATCCTCCTGCTTTTTCCCATTCATTTCTAAGTGAAGCCGTTC

<u>Primers for the construction of *mcsA* point mutants using pRSETA-*mcsA*</u>

pRSETA_Bs168_*mcsA*_R9K_for

GTGCCACGAGAAGCCAGCCACTTTTCACTTTACAAAGGTTG

pRSETA_Bs168_*mcsA*_R9K_rev

TGGCTGGCTTCTCGTGGCACTCTTGACAAATCAAGGATCC

pRSETA_Bs168_*mcsA*_R96K_for

GAAAAATTGGCAAGTTTGGATGTTCTGAATGTTACAAAACATT TC

pRSETA_Bs168_*mcsA*_R96K_rev
CATCCAAACTTGCCAATTTTTCTGAATTGCTGAAAAGTCATTCC

pRSETA_Bs168_*mcsA*_R115K_for
AATCCTAAAGAAAGTGCACAGCGGAAACACTGTGCATG

pRSETA_Bs168_*mcsA*_R115K_rev
GTGCACTTTCTTTAGGATTGGTGTAATGTTGCTATGAAATG

pRSETA_Bs168_*mcsA*_R131K_for
GATACCGAAAAAGATAGGCGGCAATCTTCATGTCAGACGGC AG

pRSETA_Bs168_*mcsA*_R131K_rev
GCCGCCTATCTTTTTCGGTATCTTACCTGCATGCACAGTGTTTC

pRSETA_Bs168_*mcsA*_R139K/

R140K_for CTTCATGTCAAGAAGCAGATTGATATGCTAAAAAAGGAATTA GAATCC

pRSETA_Bs168_*mcsA*_R139K/

R140K_rev TATCAATCTGCTTCTTGACATGAAGATTGCCGCCTATTCGTTTCG

pRSETA_Bs168_*mcsA*_R165K_for
CTCATGTAAAGGATCAAATTCGTTTATTAGAACAATCACTC

pRSETA_Bs168_*mcsA*_R165K_rev
ATTTGATCCTTTACATGAGCTGCATTTTCAAATTCTTCTTG

pRSETA_Bs168_*mcsA*_R169K_for
GATCAAATTAAGTTATTAGAACAATCACTCAAAAGTACAG

pRSETA_Bs168_*mcsA*_R169K_rev
CTAATAACTTAATTTGATCTCTTACATGAGCTGCATTTTC
