# Supplementary material for: The Involvement of the McsB Arginine Kinase in Clp-Dependent Degradation of the MgsR Regulator in Bacillus subtilis
Source: Front Microbiol. 2020 May 12;11:900. doi: 10.3389/fmicb.2020.00900 (PMC7235348; doi:10.3389/fmicb.2020.00900)
Supplement: TABLE S2 — List of used primers for MgsR mutagenesis. [file Table_2.pdf]

Table S2. List of used primers for MgsR mutagenesis

name	sequence
mgsR_+5`mRNA region_for	ACAAAGAGATGGTTAACTGAGGAGGAAAAAACGAATGGAACAACAATTAACTTTTTAC
pX_UP+mgsR 5`region_fusion_rev	CCTCAGTTAACCATCTCTTTGTGATTTAAGTGAACAAGTTTATC
amyE_front_for	TGTTTGCAAAACGATTCAAAAC
amyE_back_rev	AATGGGGAAGAGAACCGC
mgsR_R17K_for	CTGCACGTCATGCAAGAAAACAAAACATTG
mgsR_R17K_rev	CAATGTTTTGTTTTCTTGCATGACGTGCAG
mgsR_R37/42K_for	CGGCACTTATTCAAAGAAACCCCGACAAAAG
mgsR_R37/42K_rev	CTTTAATTCTTCTTTTGTCGGGGTTTCTTTG
mgsR_R33K_for	GAATTTAACGAAAAGCACTTATTCAGAG
mgsR_R33K_rev	CTCTGAATAAGTGCTTTTCGTTAAATTC
mgsR_R63K_for	GAAATTTTGGCGACAAAAAGCCAGACTTTC
mgsR_R63K_rev	GAAAGTCTGGCTTTTTGTCGCCAAAATTTC
mgsR_R94/95K_for	GAAGCCGAAGCTTTTGAAGAAACCGATTCTAGTAGACAAC
mgsR_R94/95K_rev	GTTGTCTACTAGAATCGGTTTCTTCAAAAGCTTCGGCTTC
mgsR_R33E_for	GAATTTAACGAAGAGCACTTATTCAGAG
mgsR_R33E_rev	CTCTGAATAAGTGCTCTTCGTTAAATTC
mgsR_R63E_for	GAAATTTTGGCGACAGAAAGCCAGACTTTC

mgsR_R63E_rev	GAAAGTCTGGCTTTCTGTCGCCAAAATTTC
mgsR_R94/95E_for	GAAGCCGAAGCTTTTGGAGGAACCGATTCTAGTAGACAAC
mgsR_R94/95E_rev	GTTGTCTACTAGAATCGGTTCCTCCAAAAGCTTCGGCTTC
mgsR_37/42E_for	CGGCACTTATTCGAAGAAACCCCGACAGAAG
mgsR_R37/42E_rev	CTTTAATTCTTCTTCTGTCGGGGTTTCTTCG
mgsR_R17E_for	CTGCACGTCATGCGAGAAAACAAAACATTG
mgsR_R17E_rev	CAATGTTTTGTTTTCTCGCATGACGTGCAG
mgsR_up200+_BamHI_for	CGGGATCCCCCGAGAGTCCCTCTGACTG
mgsR_BamHI_rev	GAGGATCCTCATGCAGATTGATGAACCGT
C-STREP_rev	GAGGATCCTCATTTTTCAAATTGCGGATGTGACCA
mgsR_C-STREP_to_add_rev	AAATTGCGGATGTGACCATGCAGATTGATGAACCGTTTTC
